# Supplementary material for: 53BP1-RIF1 and DNA-PKcs show distinct genetic interactions with diverse chromosomal break repair outcomes
Source: Nat Commun. 2025 Nov 24;16:10361. doi: 10.1038/s41467-025-65329-3 (PMC12644711; doi:10.1038/s41467-025-65329-3)
Supplement: Supplementary file 1 — Supplementary Information [file 41467_2025_65329_MOESM1_ESM.pdf]

Name	Purpose	Sequence (5' → 3', all sgRNA sequences have an initial G nucleotide, regardless of whether it is part of the targeted sequence)
7a (EJ7-GFP-a)	sgRNA	GACCACCCTGACCTACGGCTA
7b (EJ7-GFP-b)	sgRNA	GGCTGAAGCACTGCACGAAT
MTAP	sgRNA	GTTACCATGGTCTGTATGGCC
CDKN2B-AS1	sgRNA	GTCGTCACTACGCTGATGGCA
LMNA	sgRNA	GCCATGGAGACCCCGTCCCAG
53BP1sg1	sgRNA	GCATAATTTATCATCCACGTC
53BP1sg2	sgRNA	GAGAGAATGAGGCTCGAAGTG
RIF1sg1	sgRNA	GAAGTCTCCAACAGCGGCGCG
MAfustionHYB	primer	/56-FAM/GAAGGGAGA/ZEN/GAGAGGAGGGA/3IABkFQ/
MTAPctrlDN1	primer	TCCAGTGTCCTCAAATTCCA
MAfusion1UP	primer	AAGTGTGATGGGCAAGAAGG
MAfusion1DN	primer	AGGATTCTGCACTTGGATGG
RIF1 siRNA	siRNA	UCACGUAGCCCUAAAUUUA<br>AGACGGUGCUCUAUUGUUA<br>GUGAGGAGAUCUAAAGGUU<br>CAGAAGAGUCCAUUGCAUA

Supplementary Table 1. Oligonucleotide list.

cell line and treatment	replicate	No Indel (WT + point mutants)	Insertions	Deletions	Complex Indels	total reads
Parental 2024	1	78816	205920	110595	9436	404767
	2	94229	237897	110202	10738	453066
	3	84242	215400	101268	9190	410100
Parental M3814 2024	1	34599	136911	230191	10433	412134
	2	47869	171681	298331	13319	531200
	3	27898	106085	169215	7820	311018
53BP1-KO	1	67835	160912	95211	7033	330991
	2	100857	247787	143521	10462	502627
	3	92615	232641	134528	9618	469402
53BP1 M3814	1	17964	62505	307631	11306	399406
	2	18875	65841	292240	10451	387407
	3	15405	50907	263191	8502	338005
RIF1-KO	1	31547	87818	79130	5508	204003
	2	59527	158151	59654	7579	284911
	3	39286	106781	110532	7154	263753
RIF1-KO M3814	1	32899	222568	201689	10376	467532
	2	37897	111550	157908	9592	316947
	3	21448	73572	139332	6600	240952
PRKDC-KO 2024a	1	3002	7648	8590	461	19701
	2	1733	5017	5988	336	13074
	3	3088	7588	8283	455	19414
53BP1-KO/PRKDC-KO	1	17163	60650	145182	4795	227790
	2	17099	55894	142201	4032	219226
	3	8745	29100	74134	1888	113867
Parental repeat Dec	1	19344	48047	18857	864	87112
	2	21338	51915	18697	977	92927
	3	15265	36860	12391	611	65127
XLF-KO 2024	1	4352	8261	34027	763	47403
	2	3403	5656	26516	623	36198
	3	5537	9876	42069	1091	58573
XLF-KO/53BP1-KO	1	3433	4632	37377	1439	46881
	2	3209	5211	27626	988	37034
	3	7632	10467	59687	1645	79431

Supplementary Table 2. Read counts of EJ categories for the MA-del assay used to determine the percentages of EJ categories shown in Figures 2, 5, 7. The labels 2024/2024a/repeat Dec are used to distinguish these particular samples vs. other samples with the same cell line and treatment.

Deletion Size	µHom	MTAP Side Junction	ANRIL Side Junction	Parental DMSO			Parental M3814			53BP1-KO DMSO			53BP1-KO M3814		
				1	2	3	1	2	3	1	2	3	1	2	3
	2	GGATGCAATACAGTAGGGCCAGGC	**GC**AAGGATCATGCATGCCTTATTCA	91.96	100	100	100	100	100	100	100	100	100	100	100
-2	2	GGATGCAATACAGTAGGGCCAG**GC**	GCAAGGATCATGCATGCCTTATTCA	8.04											
	3	GGATGCAATACAGTAGGGCCAGG**C**	**GCAAGG**ATCATGCATGCCTTATTCA	39.67	34.929	37.3	48.93	46.63	47.73	46.9	48.56	47.81	47.08	48.9	47.59
	2	GGATGCAATACAGTAGGGC**CAGGC**	**GC**AAGGATCATGCATGCCTTATTCA	48.22	53.735	52.4	47.21	50.11	50.27	50.43	49.37	50.28	50.33	48.69	50.28
	2	GGATGCAATACAGTAGGGCCAG**GC**	**GCAAG**GATCATGCATGCCTTATTCA												
	0	GGATGCAATACAGTAGG**GCCAGGC**	GCAAGGATCATGCATGCCTTATTCA				2	1.86		2.16	2.07	1.91	1.7	1.87	2.13
	0	GGATGCAATACAGTAGGGCC**AGGC**	**GCA**AGGATCATGCATGCCTTATTCA												
-7	0	GGATGCAATACAGTAGGGCCAGGC	**GCAAGGA**TCATGCATGCCTTATTCA	12.11	11.336	10.3	1.86	1.4	2	0.51			0.89	0.53	
	3	GGATGCAATACAGTAGG**GCCAGGC**	**GCAAGG**ATCATGCATGCCTTATTCA				1.47					1.82		1.82	1.67
	2	GGATGCAATACAGTAGGGCCAGGC	**GCAAGGATCATGC**ATGCCTTATTCA	56.48	54.139	52.71	62.79	63.53	65.21	52.81	49.34	54.05	49.81	51.46	53.12
	2	GGATGCAATACAGTAGGGCCA**GGC**	**GCAAGGATCA**TGCATGCCTTATTCA	7.01	7.882	8.42	9.77	7.98	8.75	21.3	17.01	17.5	17.53	18.15	18.05
	1	GGATGCAATACAGTA**GGGCCAGGC**	**GCAA**GGATCATGCATGCCTTATTCA	24.42	25.153	24.59	25.98	28.48	26.05	25.88	33.65	25.14	32.66	28.57	27.16
	1	GGATGCAATAC**AGTAGGGCCAGGC**	GCAAGGATCATGCATGCCTTATTCA	7.31	6.5278	7.4									
	1	GGATGCAATACAGTAGGGCC**AGGC**	**GCAAGGATC**ATGCATGCCTTATTCA												
	0	GGATGCAATAC**AGTAGGGCCAGGC**	GCAAGGATCATGCATGCCTTATTCA	4.78	6.2979	5.31									
	0	GGATGCAATACAG**TAGGGCCAGGC**	**GC**AAGGATCATGCATGCCTTATTCA			1.58									
	0	GGATGCAATACAGTAGGG**CCAGGC**	**GCAAGGA**TCATGCATGCCTTATTCA												
-13	0	GGATGCAATACA**GTAGGGCCAGGC**	**G**CAAGGATCATGCATGCCTTATTCA									1.49			
	2	GGATGCAATACAGTAGGGCCAGGC	**GCAAGGATCATGCATGC**CTTATTCA	72.85	78.387	69.52	89.15	87.35	89.68	64.43	74.55	76.73	85.79	80.29	100
	2	GGATGCAATACAGTAGGGCCA**GGC**	**GCAAGGATCATGCA**TGCCTTATTCA	20.72	12.726	30.48	10.85	12.65	10.32	35.57	25.45	23.27	14.21	19.71	
	1	GGATGCAATACAGTAGGGCC**AGGC**	**GCAAGGATCATGC**ATGCCTTATTCA												
-17	1	GGATGCAATACAG**TAGGGCCAGGC**	**GCAAGG**ATCATGCATGCCTTATTCA	6.43	8.8865										
	4	GGATGCAA**TACAGTAGGGCCAGGC**	**GCAA**GGATCATGCATGCCTTATTCA	71.74	62.468	67.27	100	100	100	100	100	100	100	100	100
	1	GGATG**CAATACAGTAGGGCCAGGC**	**G**CAAGGATCATGCATGCCTTATTCA												
-20	0	GGATGCAATACAGTA**GGGCCAGGC**	**GCAAGGATCAT**GCATGCCTTATTCA	28.26	37.532	32.73									
	3	GGATGCAATACAGTAGGGCC**AGGC**	**GCAAGGATCATGCATGCC**TTATTCA	47.62	67.031	65.95	100	100	100	100	100	100	100	100	100
-22	2	GGATGCAATACA**GTAGGGCCAGGC**	**GCAAGGATCA**TGCATGCCTTATTCA	52.38	32.969	34.05									

Deletion Size	µHom	MTAP Side Junction	ANRIL Side Junction	RIF1-KO DMSO			RIF1-KO M3814			PRKDC-KO			53BP1-KO/PRKDC-KO		
				1	2	3	1	2	3	1	2	3	1	2	3
	2	GGATGCAATACAGTAGGGCCAGGC	**GC**AAGGATCATGCATGCCTTATTCA	100	100	100	100	100	100	100	100	100	98.29	97.47	97.63
-2	2	GGATGCAATACAGTAGGGCCAG**GC**	GCAAGGATCATGCATGCCTTATTCA										1.71	2.53	2.37
	3	GGATGCAATACAGTAGGGCCAGG**C**	**GCAAGG**ATCATGCATGCCTTATTCA	51.1	45.8	47.9	49.7	48.08	49.17	60.82	56.49	58.86			
	2	GGATGCAATACAGTAGGGC**CAGGC**	**GC**AAGGATCATGCATGCCTTATTCA	47.1	54.2	49.6	48.38	48.59	48.74	37.86	40.56	37.9	52.26	50.31	50.44
	2	GGATGCAATACAGTAGGGCCAG**GC**	**GCAAG**GATCATGCATGCCTTATTCA										1.2	1.22	1.17
	0	GGATGCAATACAGTAGG**GCCAGGC**	GCAAGGATCATGCATGCCTTATTCA	1.8		2.5	1.92	2.69	2.09	1.31	1.62	2.12			
	0	GGATGCAATACAGTAGGGCC**AGGC**	**GCA**AGGATCATGCATGCCTTATTCA										46.56	47.86	47.56
-7	0	GGATGCAATACAGTAGGGCCAGGC	**GCAAGGA**TCATGCATGCCTTATTCA					0.64			1.33	1.11		0.6	0.83
	3	GGATGCAATACAGTAGG**GCCAGGC**	**GCAAGG**ATCATGCATGCCTTATTCA	2.9	3.2	2.2	1.9		2.8	1.5	2.55	1.2	34.62	33.78	32.61
	2	GGATGCAATACAGTAGGGCCAGGC	**GCAAGGATCATGC**ATGCCTTATTCA	46.3	53.3	48.8	52.61	56.12	52.54	50.3	46.76	52.1	49.15	49.09	50.93
	2	GGATGCAATACAGTAGGGCCA**GGC**	**GCAAGGATCA**TGCATGCCTTATTCA	16.7	19.8	18	13.5	14.65	16.08	8.38	11.11	11.38			
	1	GGATGCAATACAGTA**GGGCCAGGC**	**GCAA**GGATCATGCATGCCTTATTCA	34.1	21.2	31	31.99	29.23	28.57	39.82	39.58	35.33			
	1	GGATGCAATAC**AGTAGGGCCAGGC**	GCAAGGATCATGCATGCCTTATTCA												
	1	GGATGCAATACAGTAGGGCC**AGGC**	**GCAAGGATC**ATGCATGCCTTATTCA										16.23	17.13	15.13
	0	GGATGCAATAC**AGTAGGGCCAGGC**	GCAAGGATCATGCATGCCTTATTCA		2.5										
	0	GGATGCAATACAG**TAGGGCCAGGC**	**GC**AAGGATCATGCATGCCTTATTCA												
	0	GGATGCAATACAGTAGGG**CCAGGC**	**GCAAGGA**TCATGCATGCCTTATTCA												1.34
-13	0	GGATGCAATACA**GTAGGGCCAGGC**	**G**CAAGGATCATGCATGCCTTATTCA												
	2	GGATGCAATACAGTAGGGCCAGGC	**GCAAGGATCATGCATGC**CTTATTCA	100	83.5	100	88.89	86.5	100	82.67	68.07	80.33	100	100	76.05
	2	GGATGCAATACAGTAGGGCCA**GGC**	**GCAAGGATCATGCA**TGCCTTATTCA		16.5		11.11	13.5		6.67	10.92	8.2			
	1	GGATGCAATACAGTAGGGCC**AGGC**	**GCAAGGATCATGC**ATGCCTTATTCA												23.95
-17	1	GGATGCAATACAG**TAGGGCCAGGC**	**GCAAGG**ATCATGCATGCCTTATTCA							10.67	21.01	11.48			
	4	GGATGCAA**TACAGTAGGGCCAGGC**	**GCAA**GGATCATGCATGCCTTATTCA	100	100	100	100	100	100	100	100	100			
	1	GGATG**CAATACAGTAGGGCCAGGC**	**G**CAAGGATCATGCATGCCTTATTCA										100	100	100
-20	0	GGATGCAATACAGTA**GGGCCAGGC**	**GCAAGGATCAT**GCATGCCTTATTCA												
	3	GGATGCAATACAGTAGGGCC**AGGC**	**GCAAGGATCATGCATGCC**TTATTCA	100	100	100	100	100	100	100	100	100	100	100	100
-22	2	GGATGCAATACA**GTAGGGCCAGGC**	**GCAAGGATCA**TGCATGCCTTATTCA												

Supplementary Table 3. Microhomology-associated deletions from the MA-del assay analysis. Shown for several genetic disruptions and M3814 treatments are the relative percentage of deletion sequences for individual deletion sizes. In bold/underlined text are the nucleotides deleted, and bright red text indicates deleted nucleotides showing microhomology at the EJ junction. *ANRIL* is synonymous with *CDKN2B-AS1*.

Deletion Size	µHom	MTAP Side Junction	ANRIL Side Junction	Parental DMSO			XLF-KO DMSO		
				1	2	3	1	2	3
-17	1	GGATGCAATACAG**TAGGGCCAGGC**	**GCAAGG**ATCATGCATGCCTTATTCA	**6.57**		**9.66**	**4.35**	**3.63**	**4.03**
	1	GGATGCAATACAGTAGGGCC**AGGC**	**GCAAGGATCATGC**ATGCCTTATTCA	12.80	13.40	28.50	15.53	17.43	17.49
	2	GGATGCAATACAGTAGGGCCAGGC	**GCAAGGATCATGCATGC**CTTATTCA	80.62	86.60	56.04	80.12	78.93	78.47
	0	GGATGCAATAC**AGTAGGGCCAGGC**	**GCAA**GGATCATGCATGCCTTATTCA			5.80			
-20	1	GGATG**CAATACAGTAGGGCCAGGC**	**G**CAAGGATCATGCATGCCTTATTCA	100.00	83.33	70.15	97.21	96.94	97.07
	1	GGATGCAATACAGTAG**GGCCAGGC**	**GCAAGGATCATG**CATGCCTTATTCA		16.67	29.85			
	3	GGATGCA**ATACAGTAGGGCCAGGC**	**GCA**AGGATCATGCATGCCTTATTCA				2.79	3.06	2.93
-22	2	GGATGCAATACA**GTAGGGCCAGGC**	**GCAAGGATCA**TGCATGCCTTATTCA	35.19			20.33	17.31	21.56
	3	GGATGCAATACAGTAGGGCC**AGGC**	**GCAAGGATCATGCATGCC**TTATTCA	64.81	62.50	44.62	79.67	82.69	78.44
	0	GGATGCAATACAGTAG**GGCCAGGC**	**GCAAGGATCATGCA**TGCCTTATTCA		37.50	24.62			
	0	GGATGC**AATACAGTAGGGCCAGGC**	**GCAA**GGATCATGCATGCCTTATTCA			30.77			
-23	1	GGATGCAATACAG**TAGGGCCAGGC**	**GCAAGGATCATGCATG**CCTTATTCA	100.00	31.37	55.81	49.31	45.45	50.75
	2	GGATGCAAT**ACAGTAGGGCCAGGC**	**GCAAGGAT**CATGCATGCCTTATTCA		23.53	44.19	33.68	35.80	49.25
	1	GG**ATGCAATACAGTAGGGCCAGGC**	**G**CAAGGATCATGCATGCCTTATTCA		25.49			18.75	
	1	GGATGCAATACAGTA**GGGCCAGGC**	**GCAAGGATCATGCA**TGCCTTATTCA		19.61		17.01		
-26	2	GGATGCAAT**ACAGTAGGGCCAGGC**	**GCAAGGATCAT**GCATGCCTTATTCA		100.00		53.23	59.44	56.91
	2	GGATGCAATACA**GTAGGGCCAGGC**	**GCAAGGATCATGCA**TGCCTTATTCA				46.77	40.56	43.09
-28	4	GGAT**GCAATACAGTAGGGCCAGGC**	**GCAAGGAT**CATGCATGCCTTATTCA	100.00	100.00	55.56	80.35	100.00	100.00
	0	GGATGCA**ATACAGTAGGGCCAGGC**	**GCAAGGATCAT**GCATGCCTTATTCA			44.44			
	1	GGATGCAATA**CAGTAGGGCCAGGC**	**GCAAGGATCATGCA**TGCCTTATTCA				19.65		
-30	2	GGATGCAAT**ACAGTAGGGCCAGGC**	**GCAAGGATCATGCAT**GCCTTATTCA	100.00			81.04	82.48	78.72
	2	GGATGCAATACAGTA**GGGCCAGGC**	**GCAAGGATCATGCATGCCTTA**TTCA				12.32	11.31	10.78
	1	GGATGCAATAC**AGTAGGGCCAGGC**	**GCAAGGATCATGCATGC**CTTATTCA				6.65	6.21	10.5
-31	3	GGATG**CAATACAGTAGGGCCAGGC**	**GCAAGGATCATG**CATGCCTTATTCA	100	100	100	100	100	100
-35	3	GGATG**CAATACAGTAGGGCCAGGC**	**GCAAGGATCATGCATG**CCTTATTCA	100	50		78.53	83.81	85.63
	1	G**GATGCAATACAGTAGGGCCAGGC**	**GCAAGGATCATG**CATGCCTTATTCA		50		8.32	4.55	
	2	GGATGCAATA**CAGTAGGGCCAGGC**	**GCAAGGATCATGCATGCCTTA**TTCA				13.15	11.65	14.37

Supplementary Table 4. Microhomology-associated deletions found in the XLF-KO. Shown are microhomology-associated deletions from the MA-del assay analysis that were elevated in the XLF-KO. Also shown for the parental cell line and XLF-KO are the relative percentage of each deletion sequence for individual deletion sizes. In bold/underlined text are the nucleotides deleted, and bright red text indicates deleted nucleotides showing microhomology at the EJ junction. *ANRIL* is synonymous with *CDKN2B-AS1*.

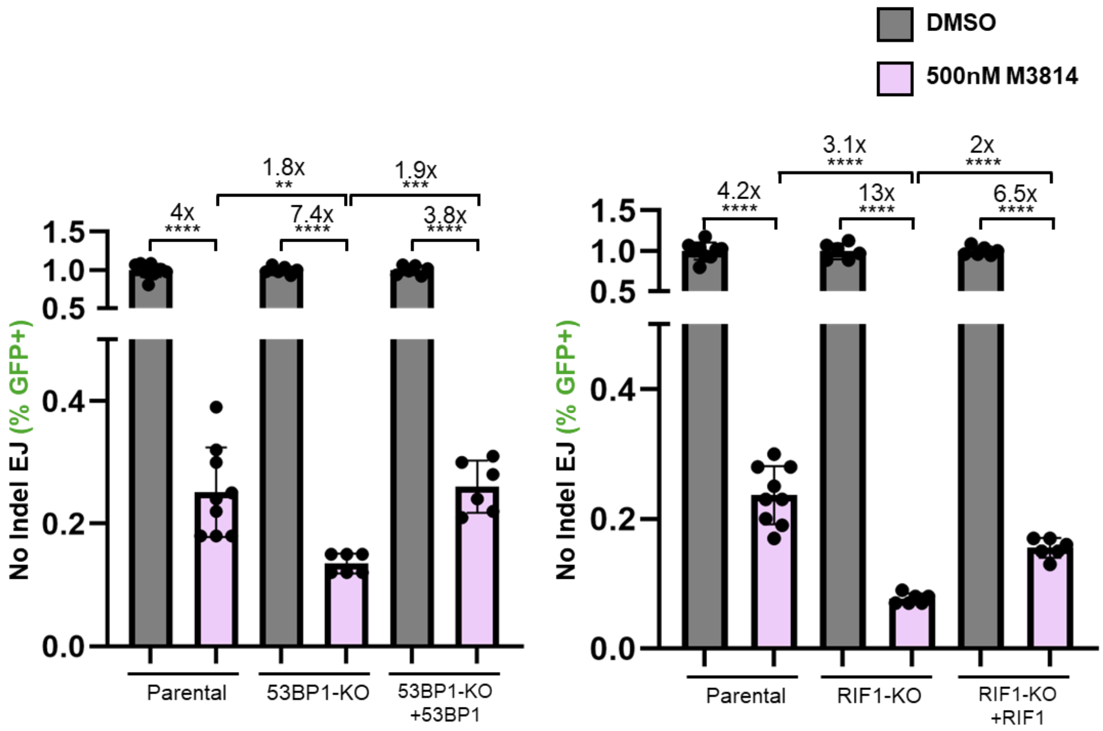

Supplementary Figure 1. Loss of 53BP1 and RIF1 magnify the effects of M3814 treatment on No Indel EJ. Shown are the EJ7-GFP data from Figures 1A and 7A, but normalized to the mean of the parallel DMSO samples for each genetic condition (mean DMSO = 1). Such normalization enables statistical analysis of the relative fold-effect of M3814 vs. DMSO for individual genetic conditions. n=6, except Parental n=9. Statistics with unpaired t-test using Holm-Sidak correction. **P>0.01, ***P>0.001, ****P>0.0001.

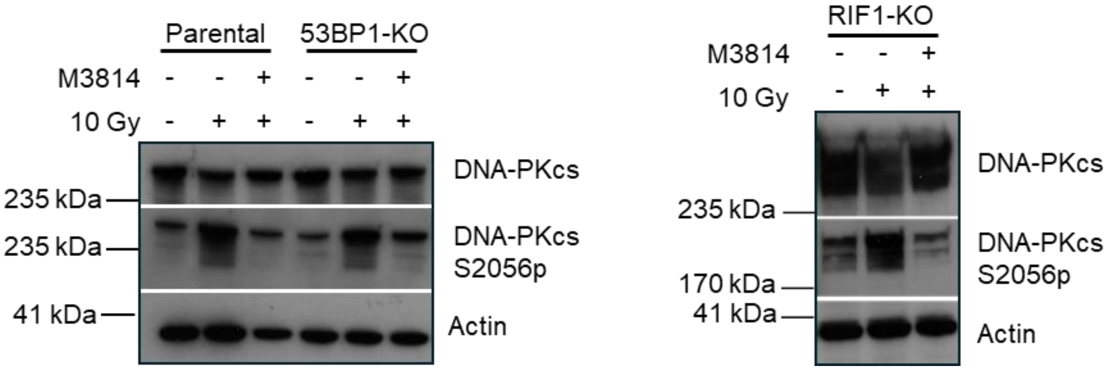

Supplementary Figure 2. M3814 inhibits DNA-PKcs autophosphorylation independent of 53BP1 and RIF1. Shown are immunoblot signals for DNA-PKcs, DNA-PKcs S2056p, and Actin from cells (Parental, 53BP1-KO, and RIF1-KO) treated with 500nM M3814 and 10 Gy IR, 10 Gy IR alone, and untreated.

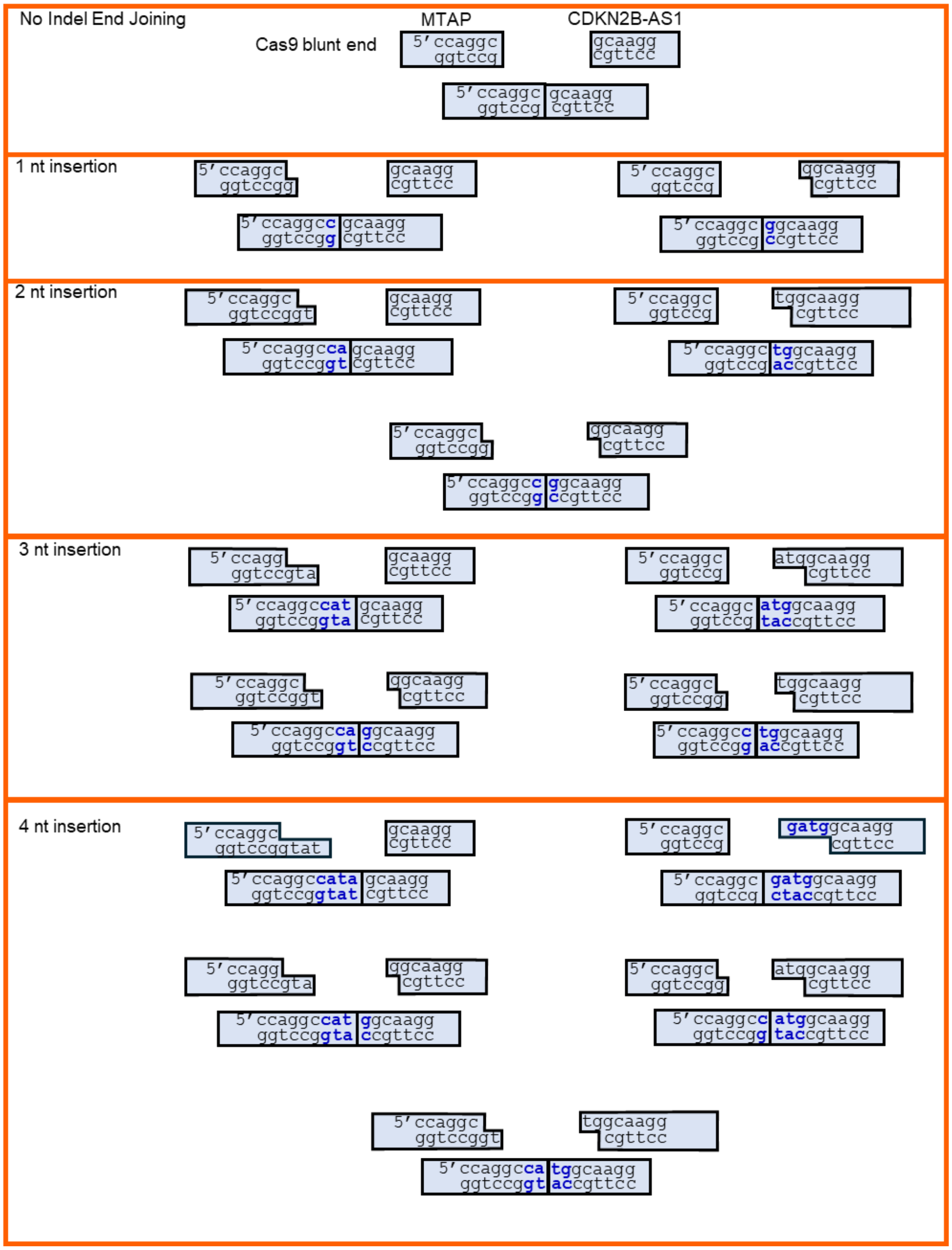

Supplementary Figure 3. Shown are diagrams of how staggered DSBs at the *MTAP* locus (left) and *CDKN2B-AS1* locus (right) in the MA-del assay could lead to various insertion sequences, which are grouped by insertion size (1-4nt).

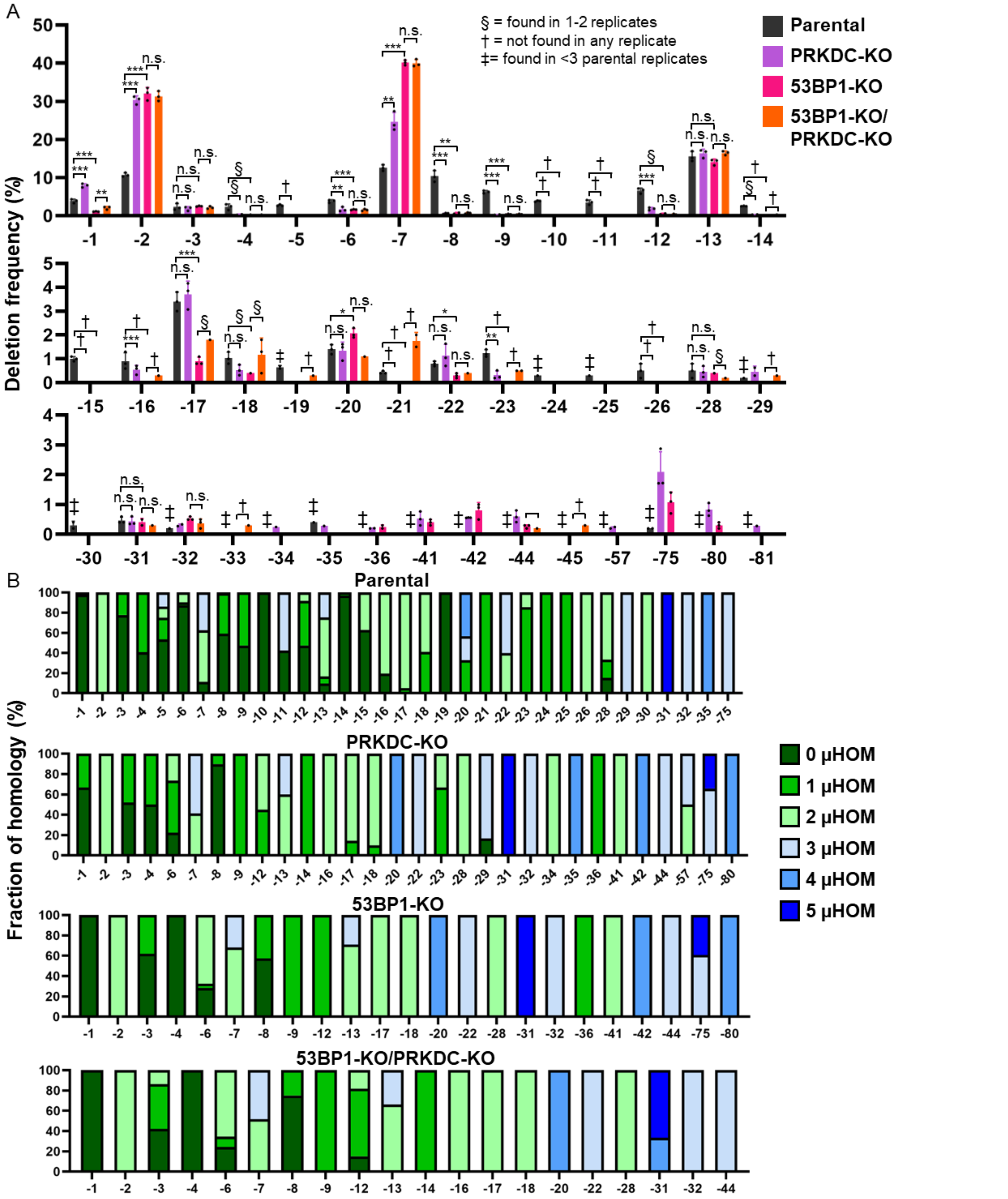

Supplementary Figure 4. Genetic loss of 53BP1 and DNA-PKcs, alone and together, cause a similar shift in deletion patterns. (A) Deletion sizes are similarly effected by genetic loss of

53BP1 and DNA-PKcs (PRKDC), and the combined disruption is similar to the single disruption. n=3 independent transfections. Statistics with unpaired t-test using Holm-Sidak correction. *P<0.05, **P<0.01, ***P<0.001, ****P<0.0001, n.s. = not significant. § = deletion size was only found in 1 or 2 replicates, † = deletion size was not found in any of the replicates, and ‡ = deletion size was found in <2 parental replicates. Parental and 53BP1-KO values are the same as those in Figure 3A. (**B)** Microhomology use varies with distinct deletions. Shown is the fraction of microhomology used for each deletion size for several experimental conditions. n=3 biologically independent transfections. Parental and 53BP1-KO values are the same as those in Figure 3B.

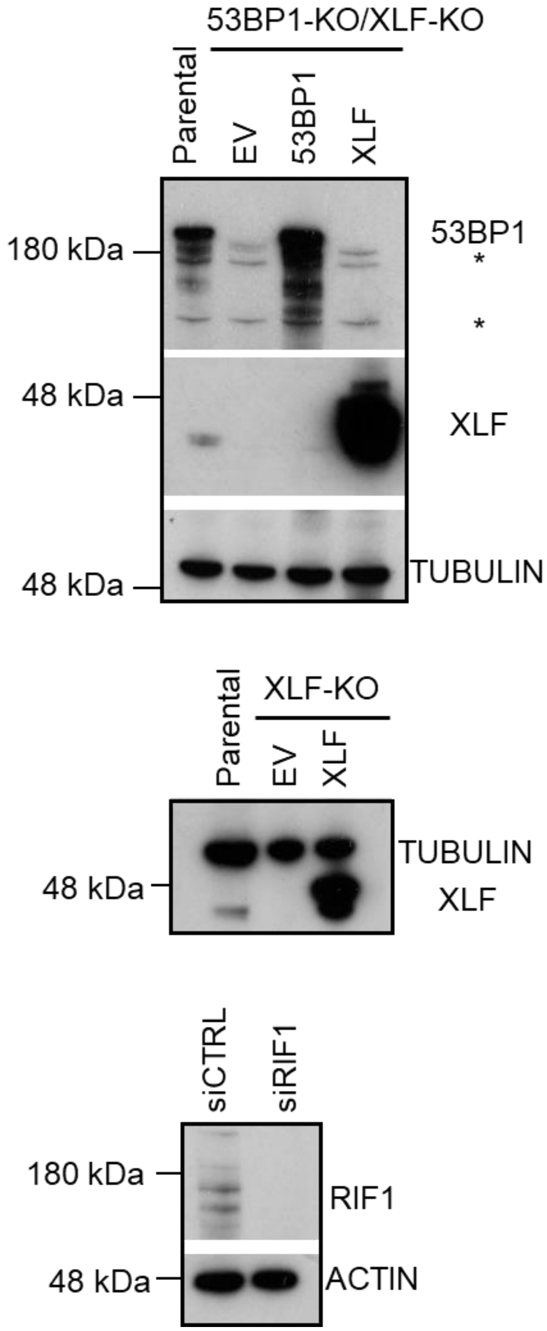

Supplementary Figure 5. Immunoblot analysis. Shown is immunoblot analysis confirming 1) expression of 53BP1 and XLF in the 53BP1-KO/XLF-KO cell line (top), 2) expression of XLF in the XLF-KO cell line (middle), and 3) depletion of RIF1 via RIF1 siRNA (siRIF1, bottom). *non-specific bands. ACTIN and TUBULIN are loading controls. Cells were transfected, protein isolated, and immunoblot analysis performed as described in the Methods.

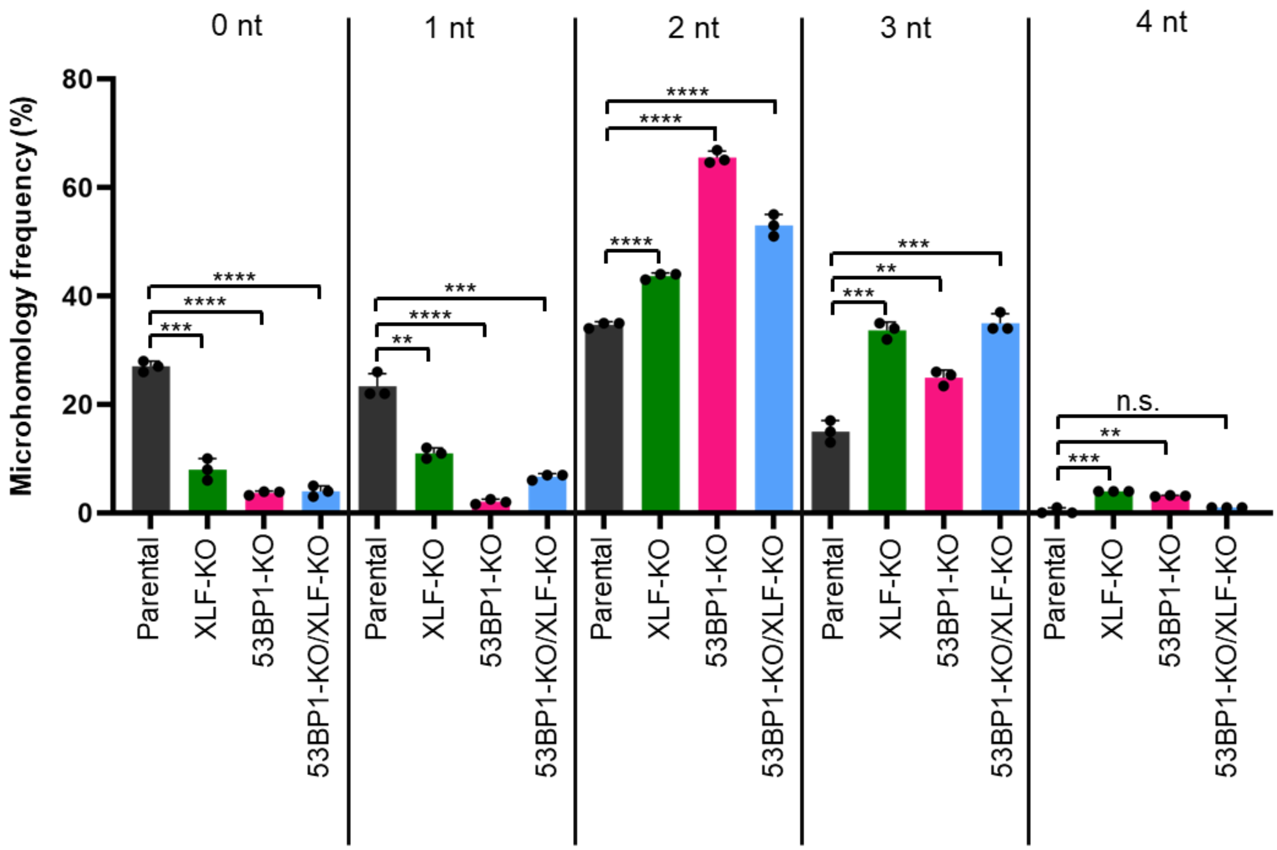

Supplementary Figure 6. Loss of 53BP1 and XLF, alone and together, cause a similar increase in microhomology usage for deletions. Shown is the frequency of microhomology used amongst deletions for each experimental condition. This is microhomology analysis of the data shown in Fig 6. n=3 biologically independent transfections. Statistics with unpaired t-test using Holm-Sidak correction. *P<0.05, **P<0.01, ***P<0.001, ****P<0.0001, n.s. = not significant.

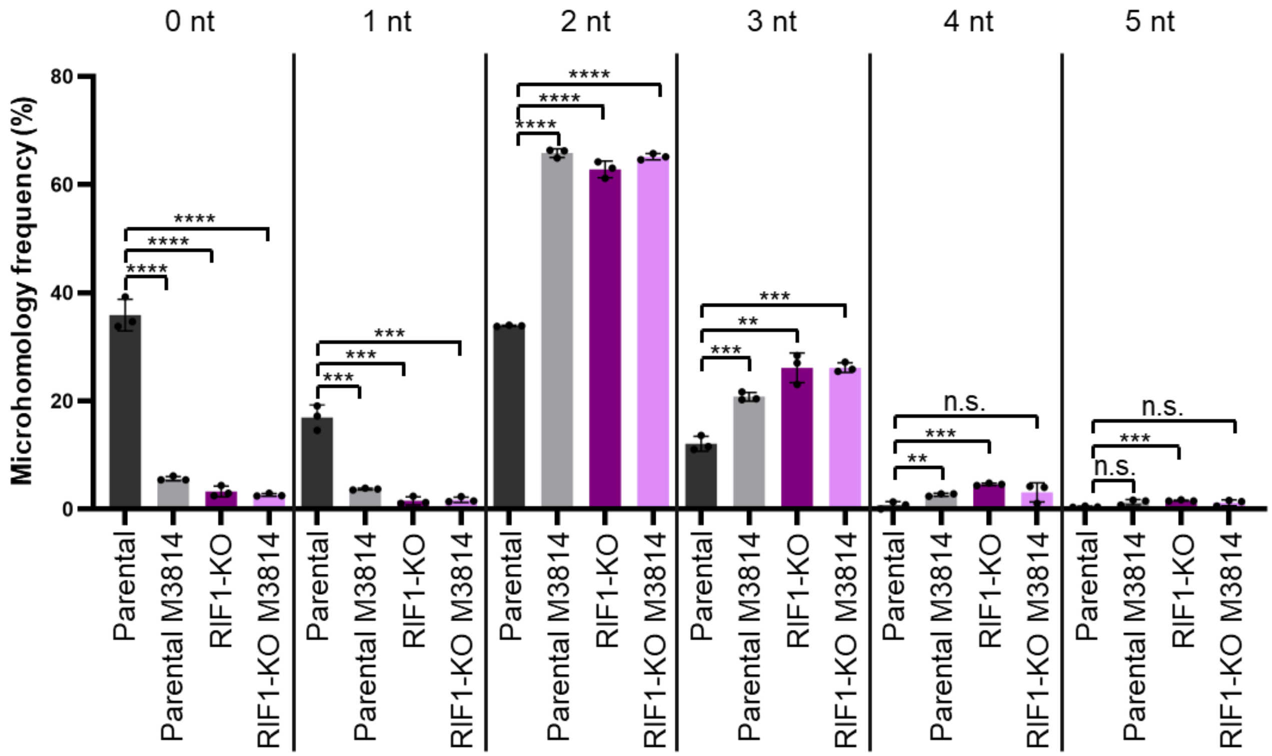

Supplementary Figure 7. Loss of RIF1 and DNA-PKcs kinase inhibition, alone and together, cause a similar increase in microhomology usage for deletions. Shown is the frequency of microhomology used amongst deletions for each experimental condition shown. This is analysis of the data shown in Fig. 8. n=3 biologically independent transfections. Statistics with unpaired t-test using Holm-Sidak correction. *P<0.05, **P<0.01, ***P<0.001, ****P<0.0001, n.s. = not significant.

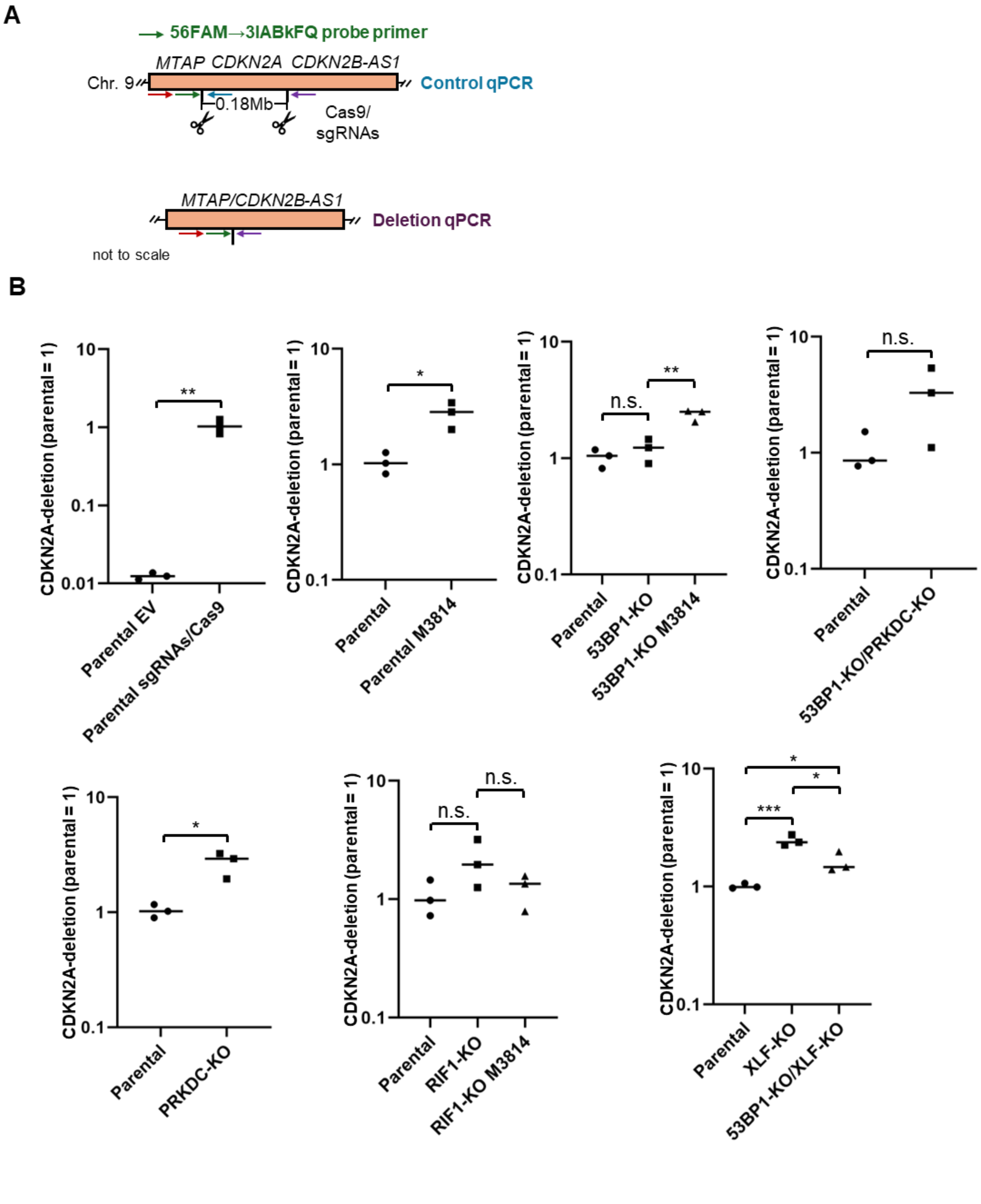

A
56FAM→3IABkFQ probe primer
MTAP CDKN2A CDKN2B-AS1
Chr. 9
Control qPCR
0.18Mb
Cas9/
sgRNAs
MTAP/CDKN2B-AS1
Deletion qPCR
not to scale
B
CDKN2A-deletion (parental = 1)
10
1
0.1
0.01
**
Parental EV
Parental sgRNAs/Cas9
*
Parental
Parental M3814
n.s.
**
53BP1-KO
53BP1-KO M3814
53BP1-KO/PRKDC-KO
PRKDC-KO
RIF1-KO
RIF1-KO M3814
***
XLF-KO
53BP1-KO/XLF-KO

Supplementary Figure 8. Genetic disruption of 53BP1, PRKDC, RIF1, XLF, and DNA-PKcs kinase inhibition (M3814 treatment) does not cause a decrease in MA-Del deletion frequency. **(A)** Schematic of the qPCR that involves three primers mapped across the MTAP break locus: the red arrow is the forward primer in *MTAP*, the green arrow denotes the 56FAM→3IABkFQ primer that is the fluorescent probe primer, the blue arrow is the reverse primer for *MTAP* (used for the control reaction), and the purple arrow is the reverse primer in *CDKN2B-AS1* (used to detect the MA-del deletion). **(B)** Deletion frequency is detectable by qPCR with the use of MA-Del Cas9/sgRNAs, and genetic disruption of the factors shown, and M3814 treatment does not obviously cause a decrease in deletion frequency. n=3 biologically independent transfections. Statistics with unpaired t-test using Holm-Sidak correction. *P<0.5, **P<0.01, ***P<0.001, n.s.=not significant.

A

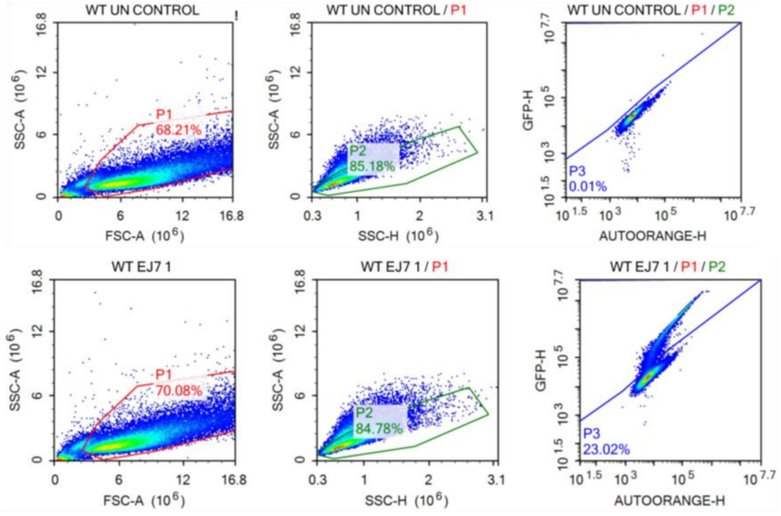

B

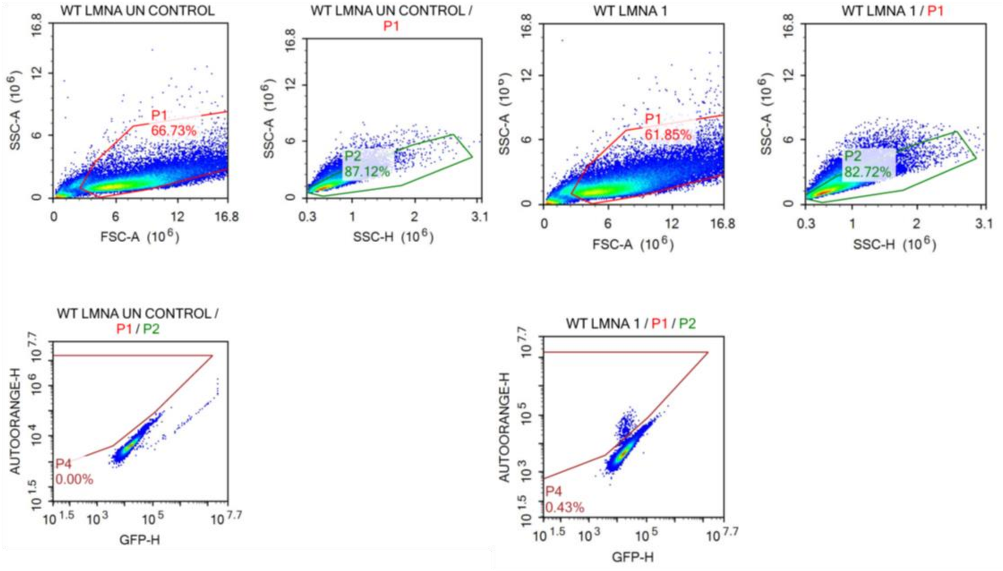

Supplementary Figure 9. Flow cytometry gating. Shown is the flow cytometry gating procedure (SSC-A/FSC-A, then SSC-A/SSC-H, then AUTOORANGE-H/GFP-H) for (A) EJ7-GFP and (B) LMNA-HDR using Parental cells as an example. Un-transfected cells are shown (labeled UN CONTROL), along with cells transfected with the relevant plasmids for the reporter assays (labeled EJ7 and LMNA 1).
