# Supplementary material for: 53BP1-RIF1 and DNA-PKcs show distinct genetic interactions with diverse chromosomal break repair outcomes
Source: Nat Commun. 2025 Nov 24;16:10361. doi: 10.1038/s41467-025-65329-3 (PMC12644711; doi:10.1038/s41467-025-65329-3)
Supplement: Supplementary file 2 — Description of Additional Supplementary Files [file 41467_2025_65329_MOESM2_ESM.pdf]

Description of Additional Supplementary Files

File Name: Supplementary Data File 1

Description: Un-analyzed alignment data for the MA-del assay for several samples:

Parental 2024 1,2,3

Parental M3814 2024 1,2,3

53BP1-KO 1,2,3

53BP1-KO M3814 1,2,3

RIF1-KO 1,2,3

RIF1-KO M3814 1,2,3

File Name: Supplementary Data File 2

Description: Un-analyzed alignment data for the MA-del assay for several samples:

PRKDC-KO 2024a 1,2,3

53BP1-KO/PRKDC-KO 1,2,3

Parental repeat Dec 1,2,3

XLF-KO 1,2,3

XLF-KO/53BP1-KO 1,2,3
